# Canine and human infection with *Borrelia burgdorferi* in the New York City metropolitan area

**DOI:** 10.1186/s13071-018-2774-z

**Published:** 2018-03-20

**Authors:** Brian H. Herrin, Melissa J. Beall, Xiao Feng, Monica Papeş, Susan E. Little

**Affiliations:** 1 Department of Veterinary Pathobiology, Center for Veterinary Health Sciences, Oklahoma State University, Stillwater, OK 74074 USA; 2 Present address: College of Veterinary Medicine, Kansas State University, Manhattan, KS USA; 3 IDEXX Laboratories, Inc., Westbrook, ME USA; 4 Institute of the Environment, University of Arizona, Tucson, AZ USA; 5 Department of Ecology and Evolutionary Biology, University of Tennessee, Knoxville, TN USA

**Keywords:** *Borrelia burgdorferi*, C6, Canine, Environmental factors, Epidemiology, Lyme disease, SNAP®4Dx® Test

## Abstract

**Background:**

Autochthonous transmission of *Borrelia burgdorferi*, the primary agent of Lyme disease in dogs and people in North America, commonly occurs in the northeastern United States, including the New York City metropolitan area, a region with a large human and pet population and broadly diverse demographics and habitats.

**Methods:**

We evaluated results from a specific, C6-based serologic assay performed on 234,633 canine samples to compare evidence of past or current infection with *B. burgdorferi* (*sensu stricto*) in dogs to county-wide social and environmental factors, as well as to reported cases of Lyme disease in people.

**Results:**

The data revealed a wide range of county level percent positive canine test results (1.2–27.3%) and human case reports (0.5–438.7 case reports/100,000 people). Dogs from highly (> 50%) forested areas and counties with lower population density had the highest percent positive test results, at 21.1% and 17.9%, respectively. Canine percent positive tests correlated with population-adjusted human case reports (*R*^2^ = 0.48, *P* < 0.0001), as well as population density, development intensity, temperature, normalized difference vegetation index, and habitat type. Subsequent multiple regression allowed an accurate prediction of infection risk in dogs (*R*^2^ = 0.90) but was less accurate at predicting human case reports (*R*^2^ = 0.74).

**Conclusion:**

In areas where Lyme disease is endemic, canine serology continues to provide insight into risk factors for transmission to both dogs and people although some differences in geographic patterns of canine infection and human disease reports are evident.

**Electronic supplementary material:**

The online version of this article (10.1186/s13071-018-2774-z) contains supplementary material, which is available to authorized users.

## Background

Lyme disease is the most common tick-borne infection reported in people in both North America and Europe [1]. In the United States, almost all (95%) human cases are reported from 13 states in the Northeast, with New York State accounting for 9.7% of reported cases [2]. Exposure to *Borrelia burgdorferi* (*sensu stricto*) in dogs, as evidenced by the presence of specific antibodies, has a similar distribution, with most infected dogs found in the northeastern region of the country. In New York State, 7.1% of pet dogs tested are seropositive [3, 4]. The eastern blacklegged tick, *Ixodes scapularis*, serves as the vector of infection to both people and dogs in this region. Infected people often develop a classic bulls-eye erythematous rash, which may be accompanied by a febrile illness that can include headache, fatigue, arthralgia and myalgia; when diagnosis and antibiotic treatment are delayed, more serious articular, cardiac and neurologic disease can develop [5]. Following dissemination of *B. burgdorferi*, dogs can also develop severe arthritis or, rarely, glomerulonephritis, although the majority of infections in dogs in North America are considered asymptomatic or subclinically infected [6, 7]. Since Lyme disease was first described in the 1970s both the geographical range of autochthonous transmission and the incidence of infection has greatly increased [8, 9]. In North America, a maintenance cycle allowing transmission is now considered to be established throughout the northeastern, midwestern, and mid-Atlantic regions of the United States, as well as in parts of southern Ontario, Quebec, Manitoba and other provinces in Canada [2, 10, 11].

Tick-borne disease risk is directly related to exposure to infected ticks; exposure may vary widely based on intensity of ticks, prevalence of infection in the local tick population, and human behaviors and habits [12]. In studies of human serology and risk factors, antibodies to *Ehrlichia chaffeensis*, another tick-borne infection in the USA, are more commonly present in individuals reporting frequent known tick exposure and those who avoid using repellents [13]. Factors significantly associated with higher risk of Lyme disease include frequent deer sightings near the home and oak habitats with ample numbers of acorns to support the rodents, which serve as reservoirs to infect ticks with the pathogen [14, 15]. Deer are a key reproductive host for adult *I. scapularis*, and several studies in North America document that, in areas where Lyme disease is endemic, higher white-tailed deer populations, measured by resident deer sightings or car accidents involving deer, are associated with an elevated risk of infection with *B. burgdorferi* [15, 16]. Infection risk is also increased in areas with high ecosystem disturbance and lower tick host diversity, presumably due to the absence of dilutional hosts. The dilution effect refers to the presence of a diverse array of vertebrates on which immature ticks feed but that do not serve as competent reservoirs for *B. burgdorferi*. This phenomenon is thought to decrease pathogen prevalence in the tick population and thus reduce overall risk of infection [17, 18]. Deciduous forests provide leaf cover that prevents ticks from desiccation and thus are also associated with increased risk, while open areas, meadows, and regions with established development generally carry a lower risk of infection [19, 20].

Several studies seeking to estimate the risk of Lyme disease have tested for the presence of the pathogens in ticks, quantitated questing ticks in the environment, and evaluated habitat factors that may influence populations of both vertebrate reservoirs and tick vectors [11, 14, 21–23]. Research using pet dogs as sentinels to document transmission of tick-borne disease agents in focused geographical areas and nationwide has been successful [24–28]. Domestic dogs inhabit the same environment as their owners and share a similar infection risk. Veterinarians throughout North America routinely test dogs for antibodies to tick-borne disease agents; reviewing the geographical and temporal patterns in the results of these tests allows identification of areas where vector-borne infections are common or increasing [3, 4, 10]. Here, we share an analysis of social and environmental factors that may contribute to risk of *B. burgdorferi* infection in dogs and compare the estimated canine infection risk to human case reports in the same region.

## Methods

**Fig. 1:**
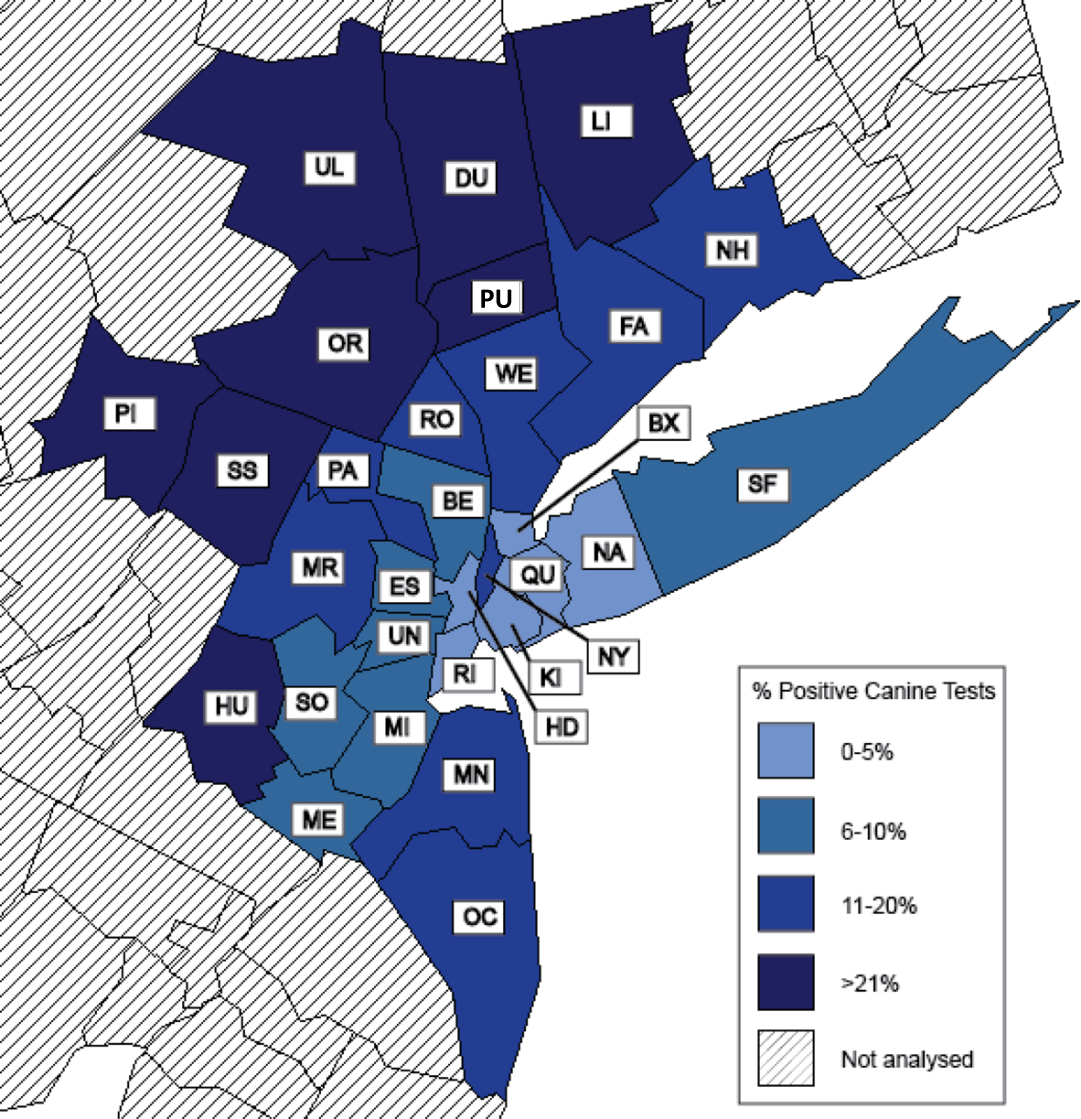
Percent positive canine tests for antibody to *Borrelia burgdorferi* by county in the New York City Metropolitan Statistical Area. Counties are labeled with 2 letter abbreviations (full names provided in Additional file 1: Table S1) and were coded as follows: 0–5% (light blue), 6–10% (blue), 11–20% (dark blue), and > 20% (very dark blue)

The study area (Fig. 1) focused on the New York City Metropolitan Statistical Area (NYC MSA) and included a total of 30 contiguous counties in New York (NY, *n* = 13), New Jersey (NJ, *n* = 13), Connecticut (CT, *n* = 3), and Pennsylvania (PA, *n* = 1). Counties and their corresponding two-letter abbreviations are provided in Additional file 1: Table S1. This region, referred to as the New York-Newark-Bridgeport Metropolitan Statistical Area, is highly interconnected and had a human population of more than 20 million by 2014 Census. When surrounding counties were also included, the entire population totaled approximately 22 million [29]. The region was selected for detailed analysis based on a number of factors, namely, large population, ample available data from testing dogs, diverse population density, and diverse environmental conditions. The region includes urban centers in New York City with high development, transitional counties with intermediate habitat types, and exurban, outer counties, that contain larger rural or forested areas. This relatively high diversity of social and environmental factors between contiguous counties allowed us to explore potential risk factors in a region where active *B. burgdorferi* transmission is known to occur to both people and dogs.

A national veterinary reporting system created and supported by IDEXX Laboratories, Inc. (Westbrook, ME, USA) provided data by county and year to allow generation of percent positive canine test results for antibodies to *B. burgdorferi*. This centralized system allowed veterinary practices screening canine patients for specific antibodies reactive to *B. burgdorferi* to submit their results. To insure anonymity of both patients and practices, all test results were grouped by location (county, state) of the reporting veterinary practices and then summed and sorted [3, 4]. Data from testing 234,633 dogs for the last decade (2001–2010) in the study area were included in the present study. All qualitative testing was conducted using in-clinic SNAP®3Dx® Test kit or SNAP®4Dx® Test kit (IDEXX Laboratories, Westbrook, ME, USA), in-clinic ELISA assays that simultaneously detect *Dirofilaria immitis* antigen and canine antibodies to *Anaplasma phagocytophilum*, *E. canis* and *B. burgdorferi*; only the *B. burgdorferi* results were used in the present study. These in-clinic assays employ a C_6_ peptide-based system to detect antibodies to *B. burgdorferi* and have been documented to have a specificity of 99.6% on field samples [24] and a sensitivity of 94.4% when compared to a combination of immunofluorescence assay (IFA) and Western blot (WB) [30]. In addition, the test used does not react to antibodies generated by vaccination [31, 32].

An initial categorical analysis was performed to determine if differences in percent positive tests results between counties were associated with general demographic and habitat types. Variables evaluated in the initial analysis included: population density [33, 34], median household income [34], percent forested area [35], percent canine samples positive for antibody to *B. burgdorferi* and annual number of human cases per 100,000 people as reported by the CDC between 2002 and 2006 [36]. All data were summarized and analyzed at the county level.

For regression, more specific environmental variables were added including precipitation and maximum and minimum temperature for the month of November. This month was selected to represent a key time in the life-cycle of *I. scapularis*, the vector present in the study area; egg deposition and larval development occurs immediately following the peak time of adult questing, mating, and feeding. Because values for temperature and precipitation show pronounced covariance, and because ticks do not have consistent reproductive activity throughout the year, the same data were not evaluated for multiple months. We downloaded 1 km resolution November minimum and maximum temperature and precipitation from PRISM Climate Group (http://www.prism.oregonstate.edu/) for 2000–2009, and calculated the averages for this period, by county. In addition, more specific land cover types replaced percent forested area used in the categorical analysis. Percent land cover types were calculated by county. The types considered included all available land cover classes in the US Geological Survey National Land Cover Database for 2006, derived from Landsat satellite imagery with 30 m resolution [37], namely: emergent herbaceous wetlands, woody wetlands, grassland/herbaceous, shrub/scrub, mixed forest, evergreen forest, deciduous forest, pasture/hay, cultivated crops, barren land and open water. To supplement population density from the categorical analysis we included intensity of development from the National Land Cover Database. Classes of development intensity provided were: high (80–100% impervious surfaces), medium (50–79% impervious surfaces), low (20–49% impervious surfaces), or no (open space, < 20% impervious surfaces). In addition, normalized difference vegetation index (NDVI) for November, averaged by county, was included as derived from Moderate Resolution Imaging Spectroradiometer (MODIS) satellite data for 2000–2009 [38].

Two-tailed Student’s t-tests were used to provide initial analysis of categorical data (StatPlus v4, AnalystSoft, Alexandria, A, USA), with significance assessed at 5% (*P* < 0.05). Variables considered were: percent positive canine tests (0–10%, 10–20%, > 20%), percent forested area (< 25%, 25–50%, > 50%), population density (< 2500, 2500–7500, > 7500 person/sq mi), human case reports of Lyme disease per 100,000 people (< 10, 10–100, > 100), and median household income (< $70,000 USD, > $70,000 USD). Variables that differed significantly were designated by different letters; variables that did not differ significantly shared the same letter designation. Regression analyses using more specific environmental data were performed (StatPlus v4, AnalystSoft, Alexandria, VA, USA), with significance assessed at 5% (*P* < 0.05). An initial simple regression was performed to compare either percent positive canine tests or human case reports to each variable. All variables significant by simple regression were analyzed pairwise using a Pearson’s correlation test; the significance of any two variables with a correlation value over 0.9 (∣ρ∣ > 0.9) was assessed and variables that did not contribute significantly to further analysis were removed [39], then multiple backward-stepwise regression was performed on remaining significant variables. Five elimination steps were performed for analysis of percent positive canine tests against social and environmental variables. Nine elimination steps were performed for analysis of human case reports.

## Results

Percent positive canine tests for *B. burgdorferi* ranged from a high of 27.3% in Putnam County (PU), NY to a low of 1.2% in Queens County (QU), NY (Fig. 1). Population-adjusted case reports of human Lyme disease ranged from a high of 438.71 case reports/10^5^ in Dutchess County (DU), NY to a low of 0.50 case reports/10^5^ in Orange County (OR), NY.

Initial evaluation of the data using only categorical values showed percent positive canine tests were significantly higher in counties with population density < 2500 persons/sq mi (17.9%, *t*_(20)_ = 2.79, *P*^AB^ = 0.01) than in counties with population density 2500–7500 persons/sq mi (8.0%) or > 7500 persons/sq mi (5.1%, *t*_(20)_ = 4.00, *P*^AB^ = 0.0007). Percent positive canine tests did not differ significantly between counties with moderate and high population density (*t*_(8)_ = 1.09, *P*^BB^ = 0.31). Population-adjusted human case reports were also significantly higher in counties with population density < 2500 persons/sq mi (113.4 case reports/10^5^, *t*_(20)_ = 2.10, *P*^AB^ = 0.05), and counties with population density 2500–7500 persons/sq mi (10.2 case reports/10^5^, *t*_(8)_ = 3.39, *P*^AB^ = 0.01) than in counties with > 7500 persons/sq mi (3.4 case reports/10^5^). No significant difference was seen in percent positive canine tests (*t*_(26)_ = 0.11, *P* = 0.91) or human case reports (*t*_(26)_ = 0.22, *P* = 0.83) between counties with median income < $70,000 (15.3%, 66.5 case reports/10^5^) and those with median income > $70,000 (13.6%, 74.9 case reports/10^5^).

Percent positive canine tests were significantly higher in counties with > 50% forested area (21.1%) than those with 25–50% forested area (15.3%, *t*_(16)_ = 2.27, *P*^BC^ = 0.037) and < 25% forested area (6.3%, *t*_(13 )_= 5.52, *P*^AC^ < 0.0001). Percent positive canine tests in counties with 25–50% forested areas were also significantly greater than those with < 25% forested area (*t*_(19)_ = 3.50, *P*^AB^ = 0.003). Population-adjusted human case reports were also significantly higher in counties with 25–50% (66.0 case reports/10^5^) or > 50% forested area (164.7 case reports/10^5^) than in counties with < 25% forested area (11.1 case reports/10^5^, *t*_(19)_ = 2.09, *P*^AB^ = 0.05; *t*_(13)_ = 3.74, *P*^AC^ = 0.003), and this trend was also seen between the moderately and densely forested counties (*t*_(16)_ = 2.29, *P*^BC^ = 0.04).

Percent positive canine tests were significantly lower in counties with < 10 human case reports/10^5^ (8.3%) than those with 10–100 human case reports/10^5^ (13.7%, *t*_(20)_ = 4.58, *P*^AB^ = 0.0002) or those with > 100 case reports/10^5^ (24.0%, *t*_(11)_ = 10.34, *P*^BC^ < 0.0001). Similarly, human case reports of Lyme disease were significantly lower in counties with < 10% positive canine test results (13.1 case reports/10^5^, *t*_(18)_ = 3.05, *P*^AB^ = 0.007; *t*_(16)_ = 5.13, *P*^AC^ = 0.0001) and counties with 10–20% positive canine test results (38.9 case reports/10^5^, *t*_(14)_ = 3.91, *P*^BC^ = 0.0016) than counties with > 20% positive canine test results (197.0 case reports/10^5^).

By simple regression, canine percent positive tests were highly positively correlated with population adjusted human case reports (*R*^2^ = 0.48, *F*_(1, 28)_ = 25.51, *P* < 0.0001). When compared to several social and environmental factors (Table 1), both canine percent positive tests and population adjusted human case reports significantly correlated with minimum and maximum temperature in November; NDVI for November; low, medium, and high-developed intensity; deciduous forest; and pasture/hay area (Table 1). Canine percent positive tests also correlated with population density, mixed forest area, and emergent herbaceous wetland, while human case reports correlated with shrub/scrub area (Table 1). Pearson’s correlation coefficient tests identified covariance between several factors, resulting in removal of November NDVI and developed high intensity area. Remaining factors that were significant for either canine percent positive tests or population-adjusted human case reports were used in subsequent multiple backward-stepwise regressions (Table 1).

**Table 1:** Significance of social and environmental variables compared to percent positive canine tests for antibodies to *Borrelia burgdorferi* (*Bb*) and human case reports of Lyme disease (LD)

Factor	Percent positive canine tests for antibodies to *Bb*			Human cases of LD/ 10^5^ population		
	*P*-value	*F*_(1, 28)_	*R* ^2^	*P*-value	*F*_(1, 28)_	*R* ^2^
Percent positive canine tests for antibodies to *Bb*	na	na	na	<0.0001	25.51	0.48
Human cases of LD/ 10^5^ population	<0.0001	25.51	0.48	na	na	na
Population density	0.0446	4.42	0.14	0.1012	2.87	0.09
Income	0.1679	2.00	0.07	0.1182	2.60	0.08
Minimum temperature (November)	<0.0001	66.36	0.70	0.0002	17.62	0.39
Maximum temperature (November)	<0.0001	34.42	0.55	0.0029	10.61	0.27
Precipitation (November)	0.1925	1.78	0.06	0.6065	0.27	0.01
Normalized Difference Vegetation Index (NDVI) (November)	0.0004	16.53	0.37	0.0126	7.11	0.20
Open water	0.1456	2.24	0.07	0.2529	1.36	0.05
Developed (open space)	0.2902	1.16	0.04	0.1226	2.54	0.08
Developed (low intensity)	0.0001	22.83	0.45	0.0009	13.65	0.33
Developed (medium intensity)	<0.0001	57.33	0.67	0.0020	11.68	0.29
Developed (high intensity)	0.0024	11.11	0.28	0.0461	4.36	0.13
Barren land	0.2547	1.35	0.05	0.4635	0.55	0.02
Deciduous forest	<0.0001	96.63	0.78	0.0003	16.94	0.38
Evergreen forest	0.2885	1.17	0.04	0.3632	0.85	0.03
Mixed forest	0.0016	12.15	0.30	0.2086	1.66	0.06
Shrub/Scrub	0.2768	1.23	0.04	0.002	11.61	0.29
Grassland/Herbaceous	0.4544	0.58	0.02	0.6411	0.22	0.01
Pasture/Hay	0.0017	12.12	0.30	<0.0001	58.80	0.68
Cultivated crops	0.1468	2.23	0.07	0.173	1.95	0.07
Woody wetlands	0.0754	3.41	0.11	0.9843	0.00	0.00
Emergent herbaceous wetlands	0.0129	7.05	0.20	0.094	3.01	0.10

*Abbreviation*: *na* not applicable

**Fig. 2:**
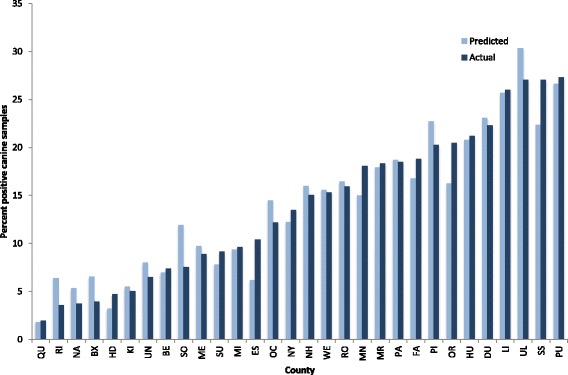
Predicted and observed percent positive canine tests for antibodies to *Borrelia burgdorferi* for each county. County abbreviations are provided in Additional file 1: Table S1

A backward-stepwise regression was calculated to predict percent positive canine tests based on 11 factors that were initially considered. After five elimination steps, remaining significant factors were human case reports per 100,000 people, population density, maximum temperature in November, deciduous forested area, mixed forest area, and precipitation in November (Table 2) resulting in a strong regression equation (*F*_(6, 23)_ = 44.76, *P* < 0.0001) with an adjusted *R*^2^ = 0.90 (Table 2). Using B values for each factor and the constant (Table 2), the predicted percent positive tests generated using the regression compared closely to the actual values reported (Fig. 2).

**Table 2:** Backward stepwise regression comparing social and environmental variables to percent positive canine tests for antibodies to *Borrelia burgdorferi. R* = 0.9597, *R*^2^ = 0.9211, adjusted *R*^2^ = 0.9005, *F*_(6, 23)_ = 44.76, *P*-level > *F* = 1.500e-11

Variable	Beta	B	*P*-level > *t*
Human case reports of Lyme disease per 10^5^ population	0.3402	0.0261	0.0002
Population density	0.2557	0.0001	0.0045
Maximum temperature (November)	0.2711	0.0182	0.0548
Deciduous forest	0.8188	31.44	5.91e-6
Mixed forest	0.3771	76.0428	0.0003
Precipitation (November)	0.2188	0.005	0.0026
Constant	-67.0655

**Fig. 3:**
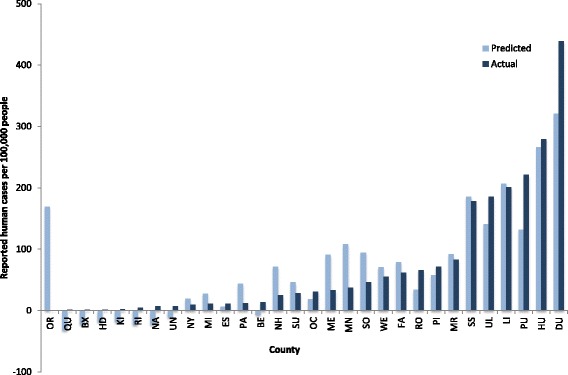
Predicted and observed human case reports of Lyme disease (LD) per 100,000 people for each county. County abbreviations are provided in Additional file 1: Table S1

For the analysis based on human case reports, 11 factors were also initially considered. After nine elimination steps, remaining significant factors were percent positive canine tests and pasture/hay area (Table 3) resulting in a less accurate predictive regression equation (adjusted *R*^2^ = 0.74, *F*_(2, 27)_ = 42.44, *P* < 0.0001). When plotted using the B values and constants (Table 3) derived from the equation, the resulting human case numbers do not closely predict reported cases (Fig. 3).

**Table 3:** Backward stepwise regression comparing social and environmental variables to human case reports of Lyme disease per 10^5^ population. *R* = 0.8710, *R*^2^ = 0.7587, adjusted *R*^2^ = 0.7408, *F*_(2, 27)_ = 42.44, *P*-level > *F* = 4.63e-09

Variable	Beta	B	*P*-level > *t*
Percent positive canine tests for antibodies to *Borrelia burgdorferi*	0.3412	4.4491	0.0055
Pasture/Hay	0.6355	1313.9	5.85e-06
Constant	-40.5891

## Discussion

The New York City metropolitan statistical area is home to approximately 6–7% of the population of the USA [29]. In general, dog populations follow human population trends; in the USA, although dog ownership varies between regions, an average of one-third of households are home to one or more pet dogs [40]. The New York City MSA proved to be an ideal region for analysis of factors contributing to risk of *B. burgdorferi* infection for a number of reasons, including: established *B. burgdorferi* transmission throughout the region; a robust dataset on canine seroprevalence consisting of 234,633 test results generated by practicing veterinarians over ten years; public availability of human case reports of Lyme disease by county for the same time period; and the presence of dramatically diverse habitat factors in close geographic proximity. This large sample size collected over a decade facilitated analysis that included consideration of a number of different social and environmental variables that are thought to contribute to the risk of *B. burgdorferi* infection. Attempts to conduct similar analyses over much larger (nationwide) geographic regions can be complicated by pronounced differences in tick phenology associated with climatic or habitat variance between regions or the number, species, and activity patterns of the most important reservoir hosts. In addition, such wide-scale analyses often include data from non-endemic areas or may be complicated by shifts in prevalence occurring in transitional zones where maintenance cycles for *B. burgdorferi* have only recently expanded [3, 4, 6].

Not surprisingly, the key factors identified as important for predicting canine infection risk in the present study (Table 2) included abiotic and biotic variables that could be involved in supporting tick populations and have been shown to be important in previous studies, such as precipitation and temperature at a key time of development of the ticks, as well as mixed forest and deciduous forest types. Similarly, those variables considered less conducive or even detrimental to tick populations such as rocky or barren land, wet habitats, or evergreen forests were not important [14, 16, 21, 41]. Appropriate temperatures and adequate precipitation combine to provide suitable humidity for ticks to thrive, while mixed and deciduous forests provide leaf litter important to shelter the ticks during egg deposition, larval hatch, overwintering, and molting of immature ticks after feeding [42]. Canine serology, however, formed the primary basis for the model in the present study, likely because the data used were survey-based and reflect cross-sectional infection risk for the canine population as a whole. Veterinarians routinely test all dogs - both healthy and sick - for evidence of antibodies indicating a past or current infection with *B. burgdorferi*.

The variables that emerged as significant for predicting human case reports of Lyme disease provided less information about the environment and habitat that should be considered highest risk for infection; indeed, the only significant factors were found to be percent positive canine tests and presence of a pasture/hay habitat (Table 3). While the presence of more pasture in a given county could reflect increased outdoor or forest-edge activity, this type of environment is not considered ideal habitat for *I. scapularis* populations [20]. However, pastures and farmland may be more prone to be converted to new housing and thus could serve as an indirect indicator of increased human presence; if edge habitat is also created, this change may elevate apparent infection risk. Overall, when compared to using canine serology generated by testing large numbers of dogs, human case reports appeared to be less accurate for identifying areas with an elevated risk of infection with *B. burgdorferi*. This difference may be due to necessary reliance on clinical or laboratory confirmation of disease in the human case report data rather than cross-sectional antibody testing, as well as variations in physician visits, patient access to medical care, and physician reporting behaviors between different communities. Similar confounding variables are thought to be contributing factors in the remarkable underestimation of the actual number of cases of Lyme disease reported each year in the USA [43].

The contribution of population density to infection risk for *B. burgdorferi* should be evaluated in light of concomitant social and environmental factors. Densely populated regions would not be expected to pose an elevated infection risk due to the presence of a largely urban, built environment. Similarly, rural, isolated areas that are not often frequented by people or dogs would be expected to appear as low risk in an analysis of this nature. Risk is created when people and dogs either reside in or enter tick habitat [12]. A “crossroads” phenomenon has been well described in which forest fragmentation resulting from roads and other anthropogenic changes that divide the forest into smaller areas increases canine and human exposure to forest edge habitat, and thereby increases exposure to ticks [44, 45]. These two competing forces cannot be addressed in the present study, but the model did show that in more densely populated areas such as Queens (QU), Bronx (BX), Hudson (HD), Kings (KI), and New York (NY) counties, there was a consistent pattern of under-prediction for both percent positive canine tests and case reports of Lyme disease in people (Figs. 2 and 3). Dogs and people in these counties appear to have a higher than expected seroprevalence of antibodies to *B. burgdorferi* or clinical presentation of disease, respectively, supporting the interpretation that many infections with this pathogen likely are acquired during travel outside the most developed, densely populated areas in the region.

Like any analysis of natural environmental predictors, the present study has a number of limitations. For example, the social and environmental factors considered in this analysis were averaged or calculated for the entire ten-year study period in an effort to minimize fluctuations that could introduce confounding bias. This approach provided a constant value for each variable considered, but also constrains the results within the historical time period evaluated. In short, the results of the present study may not accurately predict seroprevalence in dogs or human cases of Lyme disease in the future due to continued change in social and environmental variables. In addition, NDVI for November was used to reflect a key developmental time for the tick population as a whole. However, most human infections are acquired in May and June when nymphal activity peaks [7]. Finally, the spatial resolution of the analysis was limited by the fact that canine data were only available on a county level; habitat characteristics often vary widely across a given county. Available data about human cases of Lyme disease by county were also sparse and almost certainly reflect underreporting [43].

Even with the restrictions these datasets presented, we were able to use canine seroprevalence for specific antibodies to *B. burgdorferi* and several individual environmental factors to accurately predict risk of infection in an area where Lyme disease is endemic. However, this approach would likely require significant adjustment and re-evaluation prior to applying it in other regions where the phenology of tick activity may differ. Other research has shown that *I. scapularis* questing behavior differs among different populations of the tick [46], and that models in areas of ongoing emergence understandably may fail to accurately predict risk if tick populations have not yet fully established [47]. Importantly, the use of canine seroprevalence as a basis to model infection risk only has value in areas where multiple lines of evidence support the conclusion that autochthonous transmission of *B. burgdorferi* is actually occurring. Newly endemic areas are best identified by both (1) identifying the presence of infected, questing vector ticks in the environment using established, well-controlled assays, and (2) confirming specific, laboratory-based serologic evidence of transmission of that infection to people or dogs without a history of travel. In areas where Lyme disease is not endemic, the finding of dogs with antibodies reactive to *B. burgdorferi* can result from the use of less specific assays (e.g. indirect immunofluorescence assays or whole cell ELISAs), a failure to account for the possibility of a small but potentially important number of false-positives, or may stem from the inclusion of results from dogs translocated from regions where active transmission occurs [3, 4, 24, 48, 49].

## Conclusions

As documented by serologic evidence of past or current infection, dogs in the New York City metropolitan area are commonly exposed to *Borrelia burgdorferi* by *Ixodes scapularis* ticks. Most of the variation in percent positive canine tests between contiguous counties in this region can be explained by differences in habitat, precipitation, temperature, and human population density. Specific habitats that create a higher risk for infection include the deciduous and mixed forests well known to support higher *I. scapularis* populations. As expected from previous work, percent positive canine tests for *B. burgdorferi* using specific, C_6_-based assays accurately represent the risk of Lyme disease in endemic regions. Importantly, this approach would not be expected to be useful in non-endemic regions or if attempted using less specific assays. Wide scale testing of dogs for evidence of infection with the agent of Lyme disease allows insight not only into the risk faced by individual dogs, but also the tick exposure risk of the community as a whole in a way that analysis of human Lyme disease reports alone cannot.

## Additional file

Additional file 1: **Table S1**. Full names and state and county abbreviations for all counties in the New York City Metropolitan Statistical Area considered in this study. (XLSX 11 kb)

## Abbreviations

CT: Connecticut
ELISA: enzyme linked immunosorbent assay
NJ: New Jersey
NY: New York
NYC MSA: New York City Metropolitan Statistical Area
PA: Pennsylvania
